# Retraction: Oncoproteins of High-Risk HPV and EBV Cooperate to Enhance Cell Motility and Invasion of Human Breast Cancer Cells *via* Erk1/Erk2 and β-Catenin Signaling Pathways

**DOI:** 10.3389/fonc.2021.759578

**Published:** 2021-09-07

**Authors:** Frontiers Editorial Office

**Affiliations:** Frontiers Media SA, Lausanne, Switzerland

The Journal and Authors retract the 12 March 2021 article cited above for the following reasons provided by the Authors:

Following publication, concerns were raised regarding the integrity of the images in the published figures. The authors failed to provide a satisfactory explanation during the investigation, which was conducted in accordance with Frontiers’ policies.

This retraction was approved by the Chief Editors of Frontiers in Oncology and the Chief Executive Editor of Frontiers. The authors agree to this retraction.

